# Increased Cellular Senescence and Vascular Rarefaction Exacerbate the Progression of Kidney Fibrosis in Aged Mice Following Transient Ischemic Injury

**DOI:** 10.1371/journal.pone.0070464

**Published:** 2013-08-05

**Authors:** Meghan E. Clements, Christopher J. Chaber, Steven R. Ledbetter, Anna Zuk

**Affiliations:** Tissue Protection and Repair Unit, Genzyme R&D, Genzyme, a division of Sanofi, Framingham, Massachusetts, United States of America; INSERM, France

## Abstract

Recent findings indicate that elderly patients with acute kidney injury (AKI) have an increased incidence of progression to chronic kidney disease (CKD) due to incomplete recovery from an acute insult. In the current study, a co-morbid model of AKI was developed to better mimic the patient population and to investigate whether age exacerbates the fibrosis and inflammation that develop in the sequelae of progressive kidney disease following acute injury. Young (8–10 weeks) and aged (46–49 weeks) C57BL/6 mice were subjected to 30 min bilateral renal ischemia-reperfusion (I/R) to induce AKI. The aged animals have greater mortality and prolonged elevation of plasma creatinine correlating with less tubular epithelial cell proliferation compared to the young. Six weeks post-reperfusion, interstitial fibrosis is greater in aged kidneys based on picrosirius red staining and immunolocalization of cellular fibronectin, collagen III and collagen IV. Aged kidneys 6 weeks post-reperfusion also express higher levels of p53 and p21 compared to the young, correlating with greater increases in senescence associated (SA) β-galactosidase, a known marker of cellular senescence. A higher influx of F4/80^+^ macrophages and CD4^+^ T lymphocytes is measured and is accompanied by increases in mRNA of monocyte chemoattractant protein-1 (MCP-1) and tumor necrosis factor-α (TNF-α). Importantly, microvascular density is significantly less, correlating with an increase in nitro-tyrosine, a marker of oxidative stress. Collectively, these data demonstrate that prolonged acute injury in the aged animals results in an accelerated progression of kidney disease in a chronic state.

## Introduction

AKI is characterized as a sudden deterioration of renal function during which kidneys fail to excrete nitrogenous waste, maintain fluid balance and concentrate urine. It has a high rate of morbidity and mortality and is associated with long, expensive hospital stays [1], [2]. In recent years, there has been a marked rise in the incidence of AKI due in part to an increase in the aged population [2], [3], [4], [5]. As a result, finding an effective therapeutic has become an urgent need, which requires the development of better animal models that are more reflective of these patients [6], [7]. Traditional models of hypoxic kidney injury utilize young, healthy animals; however, human AKI is often accompanied by co-morbidities including aging [5], [6], [7], [8].

One of the hallmarks of AKI is the intrinsic repair that allows the kidney to recover from the injury. This occurs through a sequence of events including: the spreading and migration of surviving tubular epithelial cells that have dedifferentiated, followed by proliferation to restore cell number and re-differentiation of the epithelium [9], [10]. Recent studies have shown that incomplete repair following AKI is due to either perturbations in the cell cycle such as cell cycle arrest or defects in differentiation leading to tubular atrophy. The incomplete recovery of normal structure leads to tubulo-interstitial fibrosis, which is an important contributor to the development of CKD [11], [12].

It is well known that aging is associated with a decreased capacity to repair and regenerate damaged tissues [13], [14], [15]. In fact, the aged human population appears to be more susceptible to AKI [16] and is at higher risk for failing to completely recover from it, leading to an increased incidence of CKD [3], [5], [15], [17]. Therefore, we hypothesized that a co-morbid model of AKI with aged mice would more closely mimic the patient population with a decreased capacity to recover from AKI resulting in progressive kidney dysfunction. While previously published reports have focused on the differences in the short term effects of AKI between young and aged mice [4], [18], ours is the first study to examine progressive kidney injury in aged mice following an acute insult. Based on the greater fibrosis, inflammation, cellular senescence and vascular loss, this co-morbid model of AKI may serve as an optimal model for pre-clinical therapeutic testing and for investigating the early biological events leading to CKD.

## Experimental Procedures

### Renal I/R Injury

All studies were approved by the Genzyme Institutional Animal Care and Use Committee. Male C57BL/6 mice at ∼8–10 weeks (young) or ∼46–49 weeks (aged) of age were purchased from Taconic (Germantown, NY). They were housed in a virus- and parasite-free barrier facility with a standard 12∶12 h light-dark cycle and had ad libitum access to water and standard chow. Animals were anesthetized with sodium pentobarbital (50–70 mg/kg, ip), prepped for aseptic surgery and placed on homeothermic surgical tables (Harvard Apparatus, Holliston, MA) to maintain body temperature of 37°C through rectal probe. For some animals, the anesthesia plane was maintained with intermittent 3–5% isoflurane use of less than 5 min total duration. To induce bilateral ischemia, the renal pedicles were exposed through a flank incision, cleared of adherent connective tissue and the renal artery and vein were clamped with atraumatic microvascular clamps (Roboz, Gaithersburg, MD) for 30 min. Once the clamps were released, reflow was confirmed by visual inspection. Sham surgeries were identical without the bilateral clamp. After suturing, warm saline (1 mL) was administered by intra-peritoneal injection to maintain hydration. Mice were recovered on surgical heating pads at 37°C for 24–48 hrs. Reperfusion times included 3, 5, 24, 48, 96 hrs and 6 weeks post-ischemia. Diluted buprenorphine was administered at 0.05 mg/kg, 2.5 hrs prior to surgery and twice daily starting 24 hrs after the initial dose for a total of four doses.

### Tissue Harvesting and Measurement of Renal Function

After retro-orbital blood collection, animals were euthanized and perfused with Hank’s Balanced Salt Solution. One kidney was harvested, the kidney capsule removed and snap frozen for biochemical analysis. The second kidney was perfused with 10 mM sodium periodate-75 mM L-lysine-2% paraformaldehyde (PLP), and then immersed in it for fixation. The fixed kidney was used for all immunohistochemical analysis following paraffin or OCT embedding, as previously described [19].

Plasma creatinine and blood urea nitrogen (BUN) values were measured on Roche Integra 400 Bioanalyzer (Roche Diagnostics, IN) or a Beckman CX5 Bioanalyzer (Beckman Coulter, Inc., Brea, CA) and plotted as mean ± SD.

### Injury Score

Paraffin-embedded 5 µm kidney sections stained with hematoxylin and eosin (H&E) were blindly scored using a semi-quantitative scale of 0–5 with 0 representing no injury; 1, <20% injury; 2, 20–40% injury; 3, 40–60% injury; 4, 60–80% injury and 5, 80–100% injury. Cell exfoliation, loss of tubular architecture and formation of proteinaceous casts were evaluated in the outer stripe of the outer medulla [20], [21] while vascular congestion was predominantly found in the inner stripe of the outer medulla. Five different fields per section were scored at 20X magnification and plotted as mean ± SEM.

### Immunostaining and Histochemistry

#### Apoptosis, proliferation and oxidative stress

To identify apoptotic nuclei, frozen sections (5 µm) were probed with an ApopTag Red Apoptosis Determination kit (Millipore, Billerica, MA) according to the manufacturer’s recommendations.

To detect proliferating cells and oxidative stress, paraffin-embedded kidney sections (5 µm) were deparaffinized with EZ-Dewax solution (BioGenex, San Ramon, CA) and washed with 0.05% Tween-20 in PBS (PBS-T). Samples were quenched in 10 mM citric acid buffer containing 0.05% Tween-20, pH 6 by boiling for 30 min, then washed with PBS-T and blocked in 10% normal donkey serum. Next, samples were incubated for 1 hr at RT with primary antibody recognizing proliferating cell nuclear antigen (PCNA) for proliferation or nitrotyrosine for oxidative stress (Table 1). After washing with PBS, samples were incubated with Alexafluor 488 donkey anti-rabbit secondary antibody (25 µg/mL; Invitrogen, Carlsbad, CA) for 1 hr at RT. After extensive washing with PBS, samples were mounted with hard set mounting medium containing DAPI (Vector, Burlingame, CA) and coverslipped.

**Table 1:** Primary antibodies.

Antibody	Source	Concentration/Dilution
Rabbit anti-human PCNA	Abcam, Cambridge MA	1 µg/ml
Rabbit anti-Nitrotyrosine	Millipore, Billerica MA	1 µg/ml
Goat anti-human Collagen III	Southern Biotech, Birmingham AL	20 µg/ml
Rabbit anti-human/bovine Collagen IV	Abcam, Cambridge MA	20 µg/ml
Mouse anti-human cellular Fibronectin	Abcam, Cambridge MA	1∶200
Rat anti-mouse F4/80	AbD Serotec, Raleigh NC	20 µg/ml
Rat anti-mouse CD4	BD Biosciences, San Jose CA	0.625 µg/ml
Rat anti-mouse CD8a	BD Biosciences, San Jose CA	0.625 µg/ml
Rat anti-mouse CD19	Abcam, Cambridge MA	10 µg/ml
Rat anti-mouse CD31	Fitzgerald, Acton MA	1 µg/ml
Rabbit anti-human p21	Cell Signaling Technology, Beverly MA	1∶500
Rabbit anti-human HRP-GAPDH	Cell Signaling Technology, Beverly MA	1∶1000

#### Picrosirius red

To measure collagen deposition, an indicator of fibrosis, paraffin-embedded 5 µm kidney sections were stained with picrosirius red. Incubation time was titered to highlight staining differences in kidneys between normal mice and those from young/aged mice.

#### Extracellular matrix proteins and tissue leukocytes

Frozen sections (5 µm) were fixed in 3.2% paraformaldehyde for 10 min at RT. To immunostain for CD8a, sections instead were treated with −20°C acetone for 8 min. Next, samples were rinsed in PBS, permeabilized in 0.1% Triton X-100 and blocked in 10% normal donkey serum. Samples were then incubated overnight at 4°C with primary antibody (Table 1). After washing with PBS, samples were incubated with species-specific Alexafluor 488 or 546 donkey secondary antibody (Invitrogen) and processed as described above.

#### Senescence-associated β-galactosidase

Frozen sections (5 µm) were probed with a cellular senescence assay (Millipore, Billerica, MA) according to the manufacturer’s recommendations except for an incubation time of 1 hr in X-gal staining solution. The incubation time was titered so that endogenous β-galactosidase would not be detected.

#### Microvascular endothelial cells

CD31 (PECAM-1) was utilized as an endothelial cell marker to identify the peritubular microvasculature. Deparrafinized kidney sections were blocked with a serum-free, protein block (Dako, Carpinteria, CA) then incubated for 30 min at RT with primary antibody (Table 1). After extensive washing with 0.05% Tween-20 in TBS, samples were incubated with streptavidin-horseradish peroxidase rabbit anti-rat secondary antibody (0.4 µg/mL; Vector) for 20 min at RT. Next, samples were reacted with 3′3′-Diaminobenzidine (DAB, Vision Biosystems, Mount Waverley, Australia) for 5 min followed by rinsing and coverslipping.

### Quantitation of Immunostaining and Histochemistry

Four to six random fields within cortex and outer medulla per animal were captured at 20X magnification using a Nikon Eclipse 80i microscope (Nikon, Melville, NY). To determine the number of apoptotic and proliferating cells, Metamorph analysis was used to quantify TUNEL/PCNA positive nuclei and DAPI for total cell number. To quantify macrophage and lymphocyte markers as well as extracellular matrix proteins, total pixels per field were quantified using Adobe Photoshop® CS2 (Adobe Systems, Inc., San Jose, CA). Thresholds were determined by distinguishing background from specific signal. To quantitate β-galactosidase expression, the percentage of tubules showing positive SA β-galactosidase staining was calculated. Data are expressed as mean ± SEM.

To quantify CD31+ microvasculature and picrosirius red, whole image scans were generated at 20X magnification using the SCANSCOPE® XT scanning system from Aperio (Visa, CA). Microvessel density representing total number of CD31^+^ vessels/mm^2^ of tissue area was quantified using Aperio’s Microvessel Density Algorithm. The total area of picrosirius red stained tissue within the cortex and outer medulla and the total respective tissue area were quantified using Aperio. Final data was expressed as percent picrosirius staining. Data are plotted as mean±SEM.

### p53 ELISA

p53 expression levels were measured in whole kidney tissue homogenates using a pan p53 ELISA kit (Roche Applied Science, Indianapolis, IN) according to the manufacturer’s recommendations. Data represent mean±SD.

### Protein Extraction and Western Blot

Kidney tissues were lysed with a dounce homogenizer in ice-cold RIPA buffer (Boston BioProducts, Ashland, MA) containing 1× pefabloc, 1× protease inhibitor cocktail (Roche Applied Science, Indianapolis, IN) and 1 mM Na_3_VO_4_. After centrifugation at 14,000 rpm for 10 min at 4°C, protein concentration was measured in the supernatant by BCA assay (Pierce Biotechnology, Rockford IL). Equal amounts of protein (50 µg) were then boiled at 95°C for 5 min in 1× NuPage LDS sample buffer (Invitrogen, Carlsbad, CA) containing 0.57 M 2-mercaptoethanol (Sigma, St.Louis, MO), and separated on NuPAge Novex 4–12% Bis-Tris gels (Invitrogen). Resolved proteins were transferred to nitrocellulose membranes with an iBlot dry blotting system (Invitrogen). Membranes were then blocked with 5% (w/v) BSA in TBST (500 nM Tris, pH 7.4, 1.5 M NaCl, 0.1% (v/v) Tween-20) for 1 h at RT, and incubated overnight at 4°C with primary antibody recognizing p21 or GAPDH (Table 1). Membranes were then extensively washed in TBST and probed with goat anti-rabbit secondary antibody (Jackson ImmunoResearch Laboratories, West Grove, PA) diluted 1∶5000 for 1 hr at RT. Peroxidase was detected by SuperSignal West Dura Chemiluminescent Substrate (Pierce Biotechnology, Rockford IL) followed by exposure on Biomax XAR film (Kodak, Rochester, NY). Band intensity was quantified by densitometry with Adobe Photoshop CS6 software (Adobe Systems, Inc.) and represents mean±SD.

### Quantitative RT-PCR

Total RNA was extracted from snap frozen kidneys according to the manufacturer’s recommendation (Qiagen, Valencia, CA). RNA quality and concentration was measured at 260 nm OD. Total RNA (2 µg) was reverse-transcribed to cDNA (Clontech, Mountain View, CA) using random hexamer primers with the following cycling conditions: 42°C for 60 min, 70°C for 15 min, 4°C end. Newly synthesized cDNA then was added to TaqMan RT-PCR Mastermix (Applied Biosystems, Foster City, CA) containing primers from Applied Biosystems following standard RT-PCR reaction. Primers specific for mouse included MCP-1 (Mm99999056_m1), TNF-α (Mm00443258_m1) and 18S (Hs99999901_s1). Data are normalized to 18S and are expressed as fold change compared to normal. Data represent mean±SD.

### Statistical Analysis

Two way ANOVA followed by Bonferroni t-test was used to determine significant differences between young and aged mice. A t-test was used to determine significant differences within one age group between the normal and ischemic animals. LogRank survival analysis was used to determine differences between young and aged mice survival using SigmaStat 3.5 software (Systat Software, Point Richmond, CA).

## Results

### Aged Mice have a Greater Mortality Rate following Renal I/R Injury whereas those that Survive have a Prolonged duration of AKI which Correlates to Diminished Proliferation

**Figure 1:**
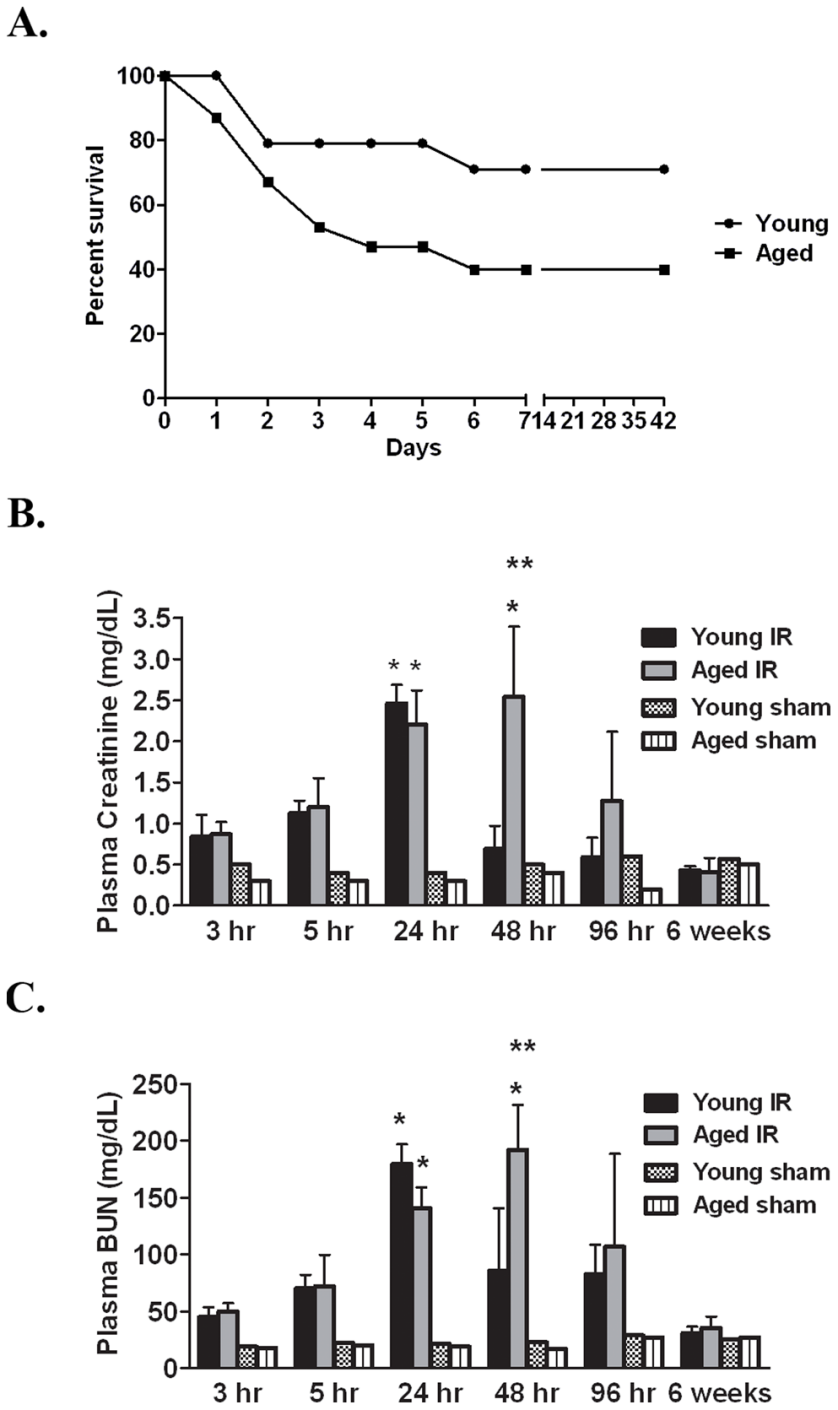
Aged mice have greater mortality and prolonged duration of AKI. A. Percent survival is greater in young mice 6 weeks post-reperfusion (n = 14–15 at day 0). Plasma creatinine (B) and BUN (C) levels were measured at indicated times post-reperfusion (n = 4–10/group) with corresponding shams that are equivalent between young and aged regardless of timepoint. *p≤0.05 indicates a significant difference from the respective sham. **p≤0.05 indicates a significant difference from the young I/R at the same timepoint.

The percent survival of human AKI is approximately 50% although this will vary depending on age, co-morbidities and severity of the injury [1]. In young mice that have undergone a 30 min bilateral renal ischemia to induce AKI, the percent survival over a six week period is 70% in contrast to 40% in aged mice, p = 0.08 (Figure 1A). All mortality occurs within the first week, with young mice reaching a plateau at 2 days post-reperfusion contrary to aged mice where death continues between 2 and 4 days post-reperfusion.

To understand the progression of kidney injury in surviving animals, time course studies were carried out to evaluate plasma creatinine and BUN. In young mice, a continual rise in plasma creatinine is measured immediately post-reperfusion with peak levels of 2.46±0.23 mg/dL at 24 hrs (Figure 1B). Kidney function returns to baseline by 48 hrs post-reperfusion (Figure 1B), confirming that young, healthy mice rapidly recover from renal I/R injury [20], [21]. Interestingly, plasma creatinine levels in aged mice reach 2.2±0.42 mg/dL at 24 hrs, which is comparable to that in the young. However, values remain significantly elevated at 2.54±0.86 mg/dL at 48 hrs (Figure 1B), reminiscent of published data in 1½–2 yrs old mice [4]. By 6 weeks post-reperfusion, renal function in the aged mice has returned to baseline. For both young and aged mice, similar trends are observed in the levels of BUN (Figure 1C). Equivalent values of plasma creatinine and BUN were measured in aged and young sham animals (Figure 1B and 1C), which are comparable to normal age matched controls (data not shown). It was not possible to measure inulin clearance for glomerular filtration rate (GFR) measurement because the poor health of aged animals within the first four days post-reperfusion precluded serial blood and urine collection.

**Figure 2:**
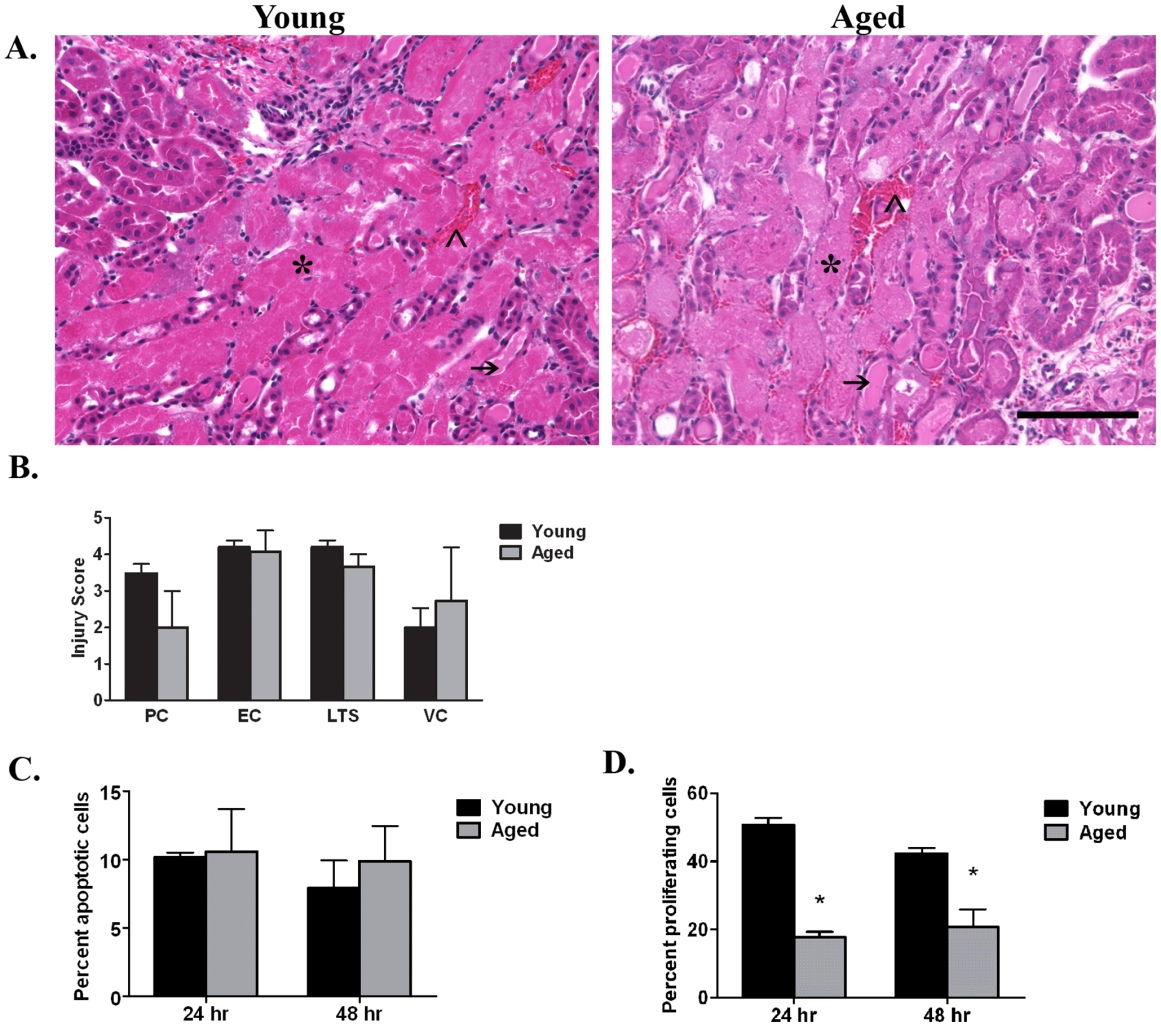
Histopathological injury and apoptosis are similar between young and aged kidneys whereas proliferation differs. A. Representative micrographs of H&E stained sections at 24 hrs post-reperfusion. Four features of injury examined: intratubular protein casts (arrow, PC), exfoliated cells (*, EC), loss of tubular structure (LTS), and vascular congestion (^∧^, VC). B. Injury score at 24 hrs (n = 8/group) post-reperfusion is similar between young and aged. C. The percent apoptotic cells is similar between the two groups as well (n = 3–4/group). D. The percent proliferation is significantly lower in the aged mice (n = 4–7/group). *p≤0.05 indicates a significant difference from young at same timepoint. No significant differences found (p≤0.05) in B and C. Bar = 100 microns.

Histopathologic analysis of H&E stained slides reveal that at 24 hrs post-reperfusion in both the young and aged mice, numerous tubules in the outer stripe of the outer medulla contain intraluminal exfoliated cells (*, Figure 2A) and/or protein casts (arrow, Figure 2A); loss of tubular integrity also is extensive. Vascular congestion is apparent (?, Figure 2A) and predominates in the inner stripe. No significant differences are observed between the two groups based on semi-quantitative scoring of histological injury (Figure 2B). Additionally, TUNEL staining indicates no significant difference in the percent apoptosis between young and aged kidneys at 24 or 48 hrs post-reperfusion (Figure 2C; compare young at 24 and 48 hrs which contain 10±0.4% and 8±2% apoptotic nuclei, respectively, to aged kidneys which contain 10±3% apoptotic nuclei at both 24 and 48 hrs post-reperfusion). TUNEL positive nuclei were <0.5% in young and aged sham kidneys (data not shown).

In contrast, cell proliferation is significantly greater in young kidneys following renal I/R. At 24 hrs post-reperfusion, kidneys from young mice have an approximately three-fold difference in the percentage of PCNA positive nuclei in the outer medulla as compared to the aged (50.7±2.1% vs 17.8±1.5%, Figure 2D). At 48 hrs, an approximately two-fold difference is measured (42.2±1.6% PCNA positive cells in the young in contrast to 20.8±5.1% in the aged kidneys; Figure 2D). Collectively, the data indicate that aged animals have a greater mortality rate and a prolonged loss in renal function post-reperfusion, correlating with decreased cell proliferation; renal histological injury and cell death, however, are comparable.

### Tubulo-interstitial Fibrosis is Greater in Aged Mice 6 Weeks after Reperfusion

**Figure 3:**
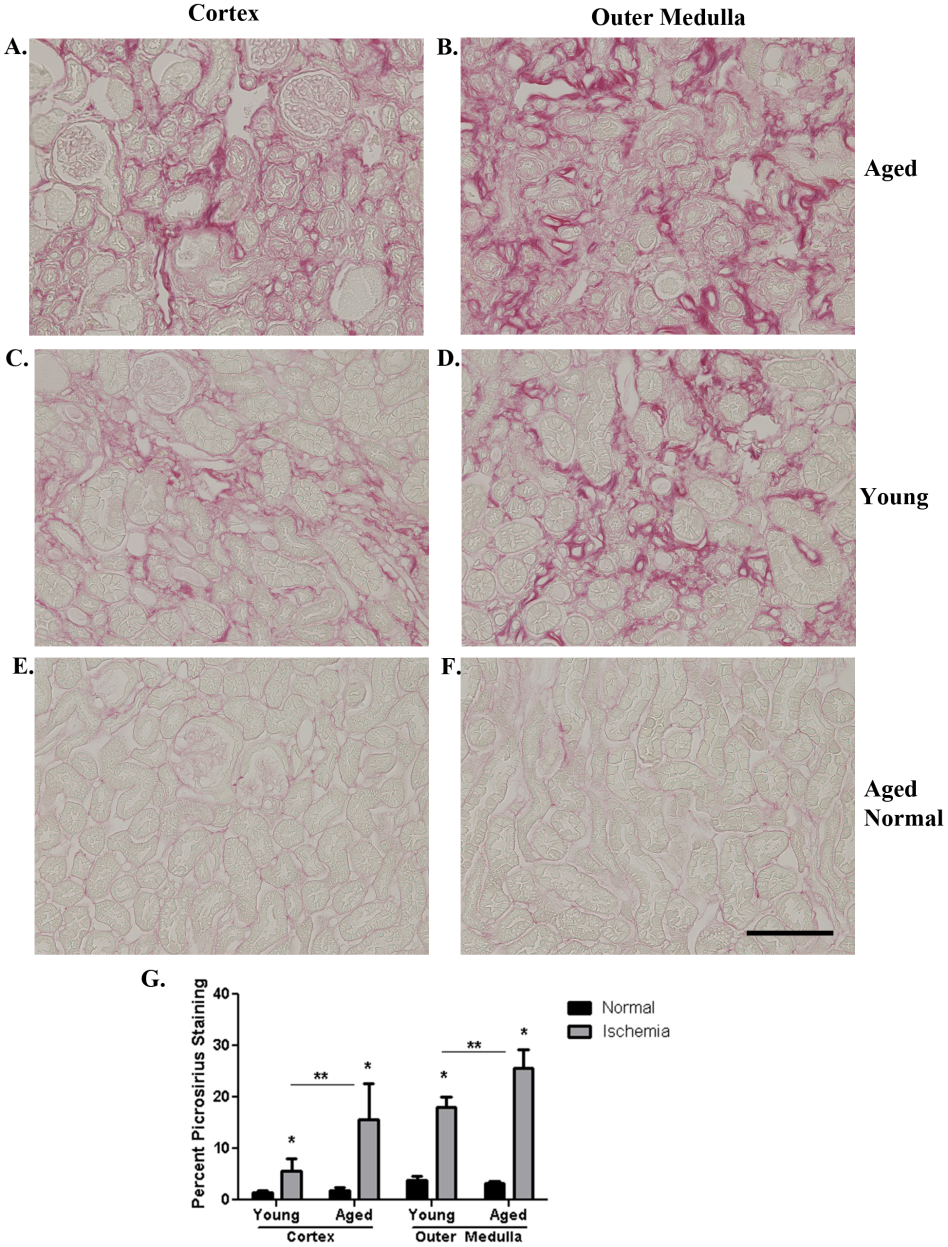
Fibrosis is greater in aged kidneys 6 weeks post-reperfusion. Representative micrographs of picrosirius red stained sections at 6 weeks post-reperfusion in the aged (A) and young (C) cortex as well as the aged (B) and young (D) outer medulla. The aged (E, F) and young (G) normal kidney have no interstitial fibrosis. Quantitation of percent picrosirius red staining (G) indicates significantly greater amounts in both the cortex and outer medulla of aged mice 6 weeks post-reperfusion. *p≤0.05 indicates a significant difference from respective normal. **p≤0.05 indicates a significant difference from young I/R. Bar = 100 microns.

To examine the long term outcomes of AKI in young and aged mice, sections stained with picrosirius red which identifies collagen were examined 6 weeks post-reperfusion. At this time, renal function measured by plasma creatinine (Figure 1B) and BUN (Figure 1C) has returned to baseline. Aged kidneys, however, have significantly more interstitial fibrosis than the young in both the cortex and outer medulla post-reperfusion (Figure 3G). Histologically, a smaller percentage of interstitial fibrosis is seen in cortex of young kidneys (5.7±2.4%, Figure 3G and 3C), becoming more extensive with aging (15.7±6.9%, Figure 3G and 3A). In the outer medulla of both young and aged kidneys, large areas of interstitial fibrosis are prevalent (18±2%, Figure 3G and 3D and 25.6±3.6%, Figure 3G and 3B, respectively), but again fibrosis is greater in the aged. In contrast, normal aged kidneys have no interstitial fibrosis (Figure 3E and F), similar to normal young (Figure 3G). Differences in extracellular matrix deposition between young and aged kidneys 6 weeks post-reperfusion were further evaluated.

**Figure 4:**
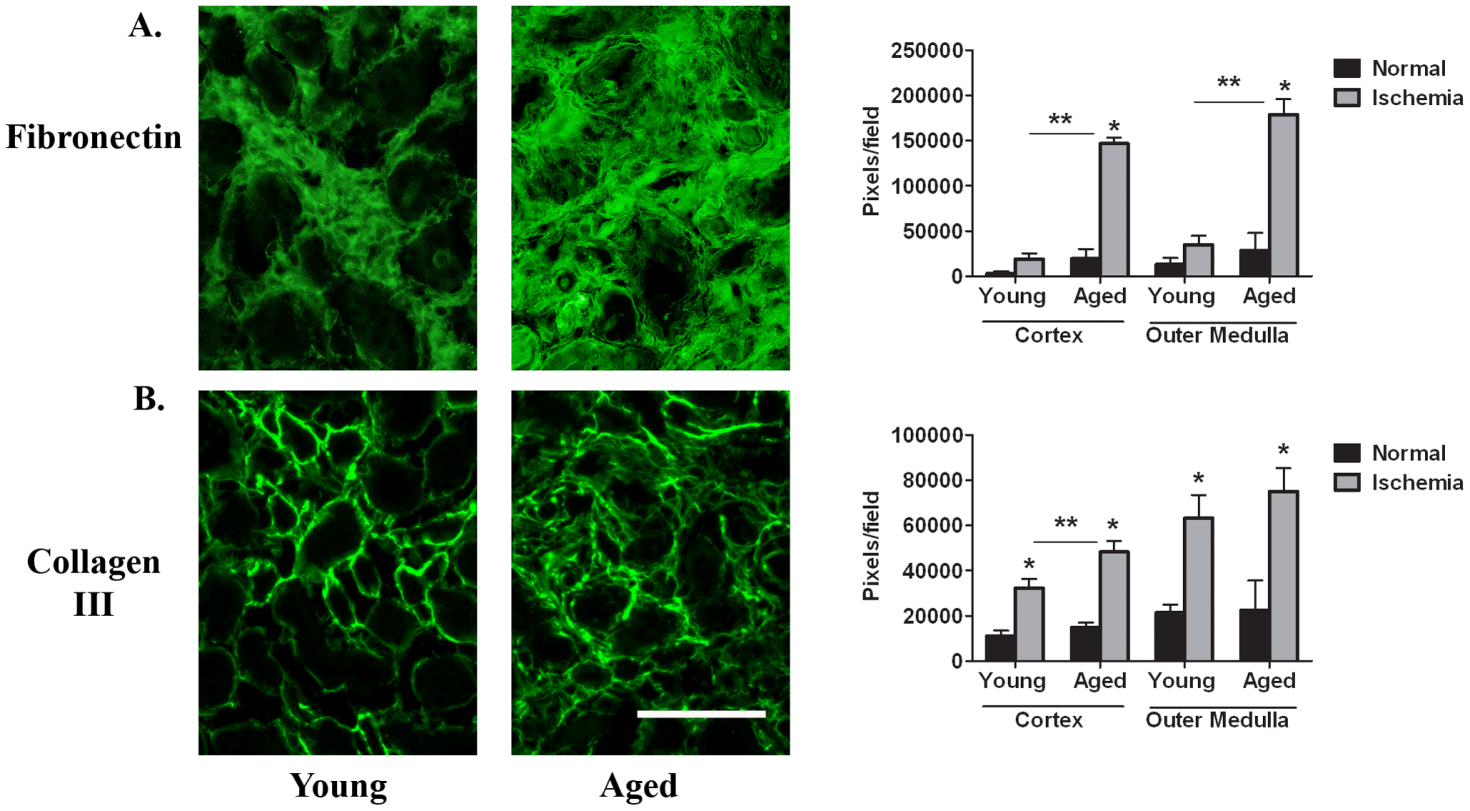
Extracellular matrix proteins are greater in aged kidneys compared to young at 6 weeks post-reperfusion. Representative micrographs of cellular fibronectin (A) and collagen III (B) immunostained sections of the outer medulla of young and aged kidneys. Quantitation of pixels per field indicates significantly increased matrix deposition in the aged kidneys 6 weeks post-reperfusion. (n = 4–5/group). There are equivalent low levels in young and age matched normals. *p≤0.05 indicates a significant difference vs respective normal. **p≤0.05 indicates a significant difference between young and aged ischemia. Bar = 100 microns.

Cellular fibronectin, an extracellular glycoprotein believed to be the first matrix molecule deposited in renal interstitial fibrosis [22], is expressed in the interstitium 6 weeks post-reperfusion (Figure 4A). Significantly higher levels are measured in aged kidneys in the cortex (146766.1±6750.1 pixels/field) and outer medulla (178200.7±17221.6 pixels/field) in contrast to the young (cortex, 19076.7±6546.3 pixels/field; outer medulla, 34583.7±10359.8 pixels/field). In normal young and aged matched kidneys, no difference in fibronectin expression is measured. Collagen III, a known fibrotic marker, is also detected (Figure 4B), with significantly greater levels quantitated 6 weeks post-reperfusion in the cortex of aged kidneys compared to the young (48166±4984.0 pixels/field vs 32156.3±4227.6 pixels/field). Equivalent expression levels in the outer medulla are found at this time in both young (63150.7±10143.0 pixels/field) and aged (74781.6±10428.9 pixels/field). Normal young and aged matched kidneys show no difference in collagen III expression in either the cortex or outer medulla. Similar data are measured for collagen IV, a known constituent of tubular basement membranes (data not shown), which also appears in the interstitial space following renal injury [19].

### Senescence Associated β-galactosidase Positive Cells Accumulate in the Fibrotic Kidney 6 Weeks Post-reperfusion, with a Greater Number in the Aged Kidney following AKI

**Figure 5:**
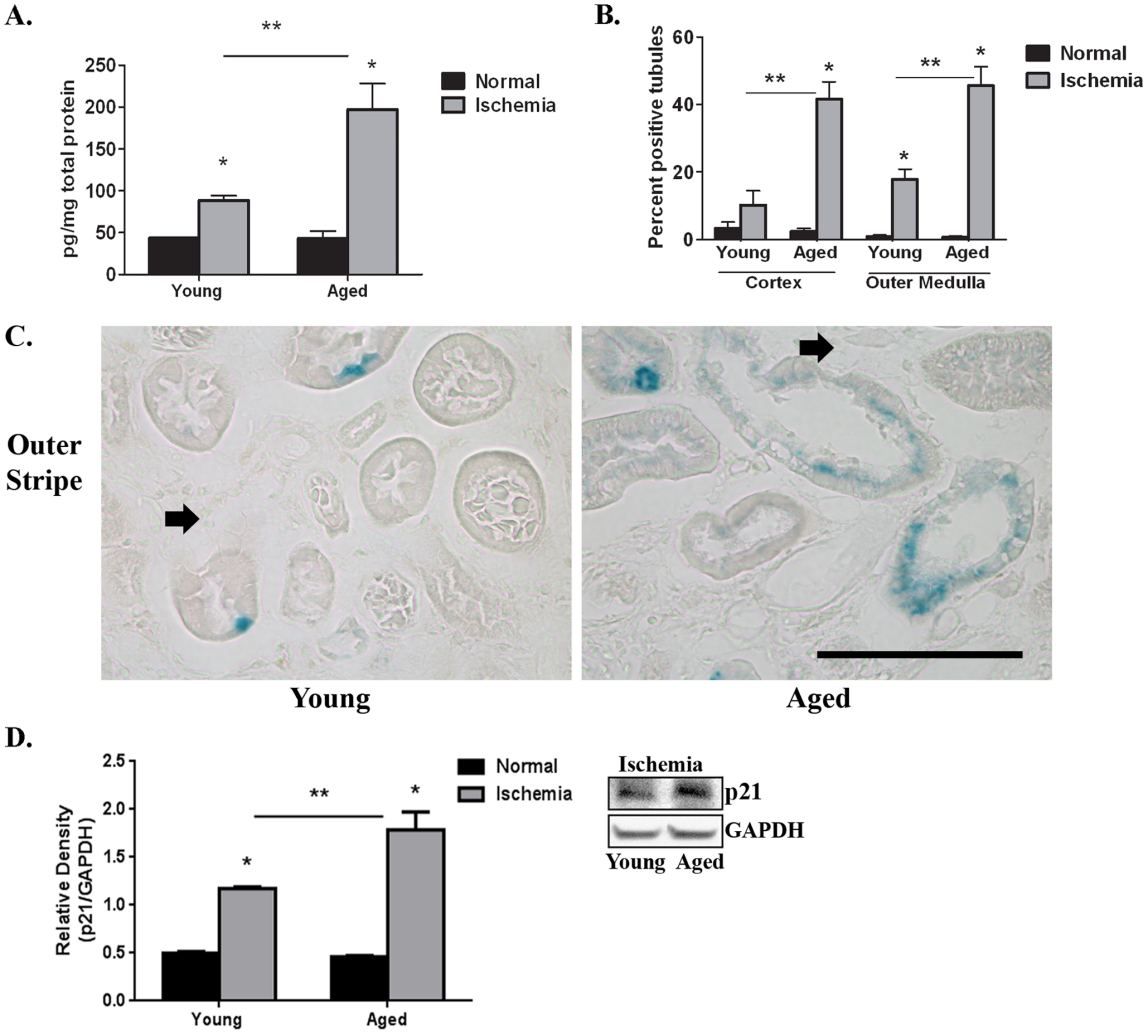
Expression of p53 and β-galactosidase is greater in aged kidneys at 6 weeks post-reperfusion. Equivalent low levels are expressed in young and age matched normals. A. p53 expression levels measured by ELISA 6 weeks post-reperfusion (n = 4–6/group). B. Quantitation of percent β-galactosidase positive tubules (n = 4–5/group). *p≤0.05 indicates a significant difference vs respective normal. **p≤0.05 indicates a significant difference between young and aged ischemia. C. Representative micrographs of frozen sections of β-galactosidase staining in the outer stripe at 6 weeks post-reperfusion. Some tubules that are β-galactosidase positive are adjacent to fibrotic areas (arrow). Bar = 100 microns. D. Quantification of Western blots for p21 normalized to GAPDH in kidney tissue (n = 3/group) with a representative blot. *p≤0.05 indicates a significant difference between young and aged ischemia.

Since a p53-mediated cell cycle arrest promotes the development of fibrosis following AKI [12], we evaluated p53 levels 6 weeks post-reperfusion (Figure 5A). In young and aged matched normal kidneys (Figure 5A), p53 expression is equivalent (43±0.45 vs 43±9 pg/mg total protein). However, there is a significant increase 6 weeks post-reperfusion in young mice (88.34±5.68 pg/mg total protein) compared to its respective age matched normal (43±0.45 pg/mg total protein; Figure 5A). Importantly, there is a significantly greater rise in p53 levels in the aged mice 6 weeks post-reperfusion (197.4±30.6 pg/mg total protein) compared to the young 6 weeks post-reperfusion (88.34±5.68 pg/mg total protein; Figure 5A).

In addition to regulating cell cycle progression, the p53 signaling pathway has been linked to both apoptosis and cellular senescence [23]. However, no difference in apoptosis could be measured by TUNEL staining between young and aged kidneys 6 weeks post-reperfusion with <0.5% apoptosis (data not shown). However, immunohistochemistry for β-galactosidase, a widely accepted marker of cellular senescence [24], reveals significantly more β-galactosidase positive tubules in the cortex and outer medulla of aged kidneys 6 weeks post-reperfusion compared to the young (Figure 5B; compare aged, cortex, 41.5±5.8% positive tubules to young, cortex, 10.1±4.3% positive tubules and aged, outer medulla, 45.52±5.7% positive tubules to young, outer medulla, 17.8±2.9 positive tubules). There is also significantly greater staining in the cortex of aged mice (41.5±5.8% positive tubules) compared to its respective aged matched normal (2.4±0.9% positive tubules; Figure 5B). However, in young mice 6 weeks post-reperfusion, the percentage of β-galactosidase positive tubules is not significantly different from young normal (compare 10.1±4.3% to 3.3±2%, respectively; Figure 5B). In the outer stripe of the outer medulla, both the young (17.8±2.9% positive tubules) and aged (45.52±5.7% positive tubules) mice 6 weeks post-reperfusion have significant increases above normal (0.9±0.4% positive tubules; Figure 5B).

SA β-galactosidase localizes primarily to proximal tubular epithelia, with some tubules bordering areas of fibrosis (arrow, Figure 5C). Not all cells within the tubule are immunoreactive, highlighting cellular differences in the response to renal I/R injury. β-galactosidase positive cells also are sometimes seen in the distal nephron (data not shown); however, glomeruli and perivascular cells are not immunoreactive.

There was enhanced activation of the p53 pathway determined by measuring expression of p21, a downstream target [25] activated by direct DNA binding of p53 to the promoter. Densitometry of Western blot analysis indicates a statistically significant increase of p21 in aged mice 6 weeks post-reperfusion compared to the young 6 weeks post-reperfusion as well as age-matched normals (Figure 5D). Since the p53-p21 axis is known to regulate cellular senescence [25], these data support our novel observation that following I/R injury, aged kidneys have more β-galactosidase positive senescent cells.

### More Inflammatory Cells Infiltrate the Aged Kidney 6 Weeks Post-reperfusion

**Figure 6:**
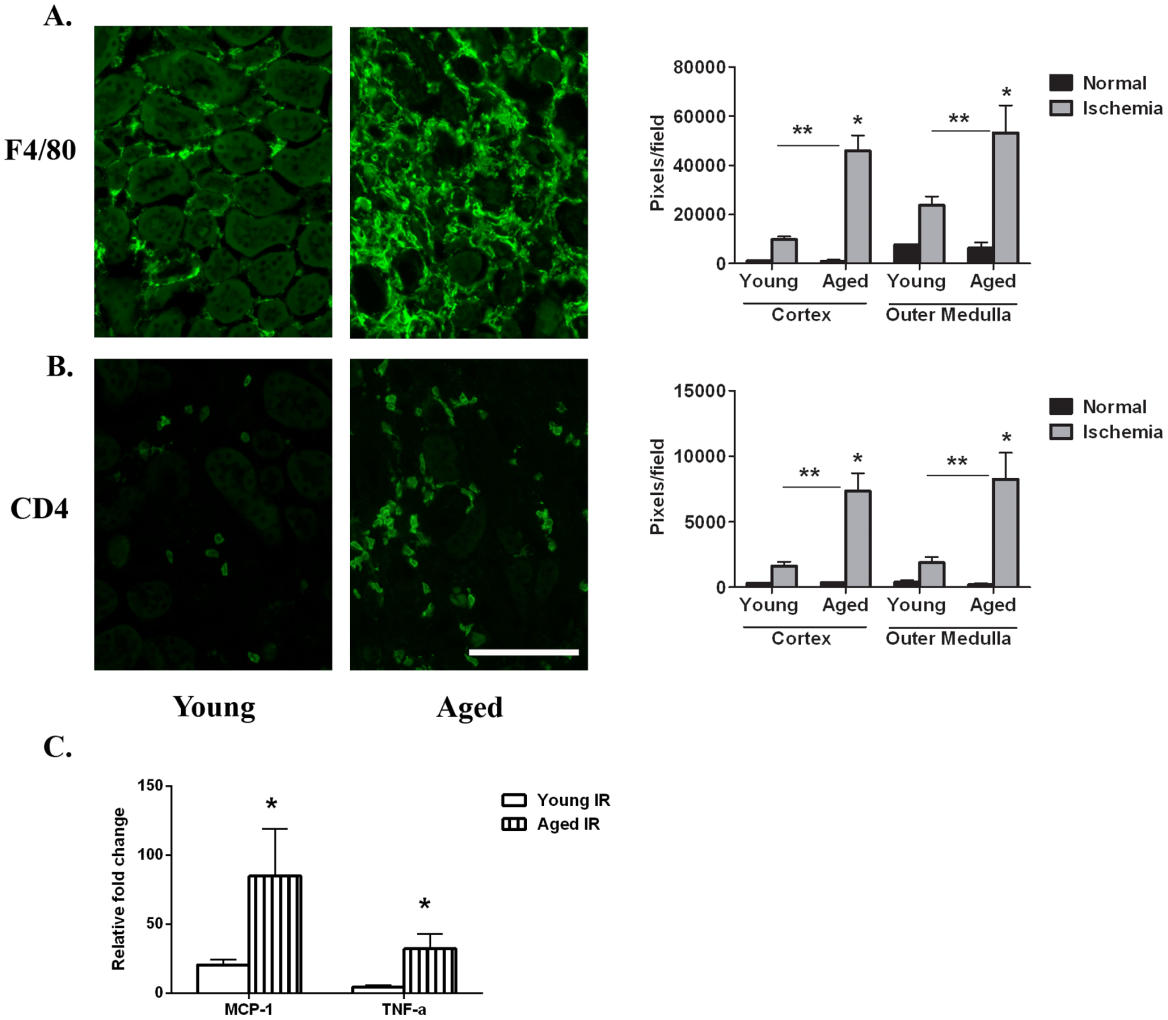
Inflammation in the cortex and outer medulla 6 weeks post-reperfusion is greater in aged kidneys. Representative micrographs of F4/80 (A) and CD4 (B) immunostained sections of the outer medulla of young and aged kidneys. Quantitation of pixels per field indicate significantly increased numbers of cells in the aged kidneys 6 weeks post-reperfusion (n = 4–5/group). There are equivalent low levels in young and age matched normals. *p≤0.05 indicates a significant difference vs respective normal. **p≤0.05 indicates a significant difference between young and aged ischemia. Bar = 100 microns. C. Gene expression of MCP-1 and TNF-α indicate increased mRNA levels in the aged kidneys 6 weeks post-reperfusion (n = 7 for young and 3 for aged mice). *p≤0.05 indicates a significant difference between young and aged ischemia.

Persistent inflammation following AKI has been associated with long term changes including fibrosis [11], [26], [27]. The F4/80^+^ macrophage is the most abundant leukocyte in the kidney 6 weeks post-reperfusion regardless of age (Figure 6A). However, significantly more F4/80^+^ macrophages are present in the cortex and outer medulla of aged kidneys in contrast to the young following AKI (aged vs young: cortex, 45825.7±6332.9 pixels/field vs 9713.3±1324.2 pixels/field; outer medulla, 53137.4±11238.8 pixels/field vs 23687.1±3599.9 pixels/field; Figure 6A). Similarly, the aged animals have a greater number of infiltrating lymphocytes. CD4^+^ T lymphocytes (Figure 6B) are identified between tubules in the interstitium of the cortex and outer medulla of young kidneys 6 weeks post-reperfusion with larger amounts in the aged at this timepoint (aged vs young: cortex, 7324.5±1381.6 pixels/field vs 1630.6±317.7 pixels/field; outer medulla, 8225.5±2025.8 pixels/field vs 1867.3±447.9 pixels/field). CD8^+^ T lymphocytes also are found at higher levels in the aged kidney (aged vs young: cortex, 2227±226 pixels/field vs 715.1±323.5 pixels/field; outer medulla, 2994.8±694.4 pixels/field vs 631.1±98.1 pixels/field). In contrast, CD19^+^ B lymphocytes are absent (data not shown). Normal young and aged matched kidneys contain equivalent low numbers of leukocytes (Figure 6A+B). Consistent with greater numbers of macrophages and T lymphocytes in aged kidneys post-reperfusion, mRNA expression of MCP-1 and TNF-α are also increased 6 weeks following I/R injury (Figure 6C).

**Figure 7:**
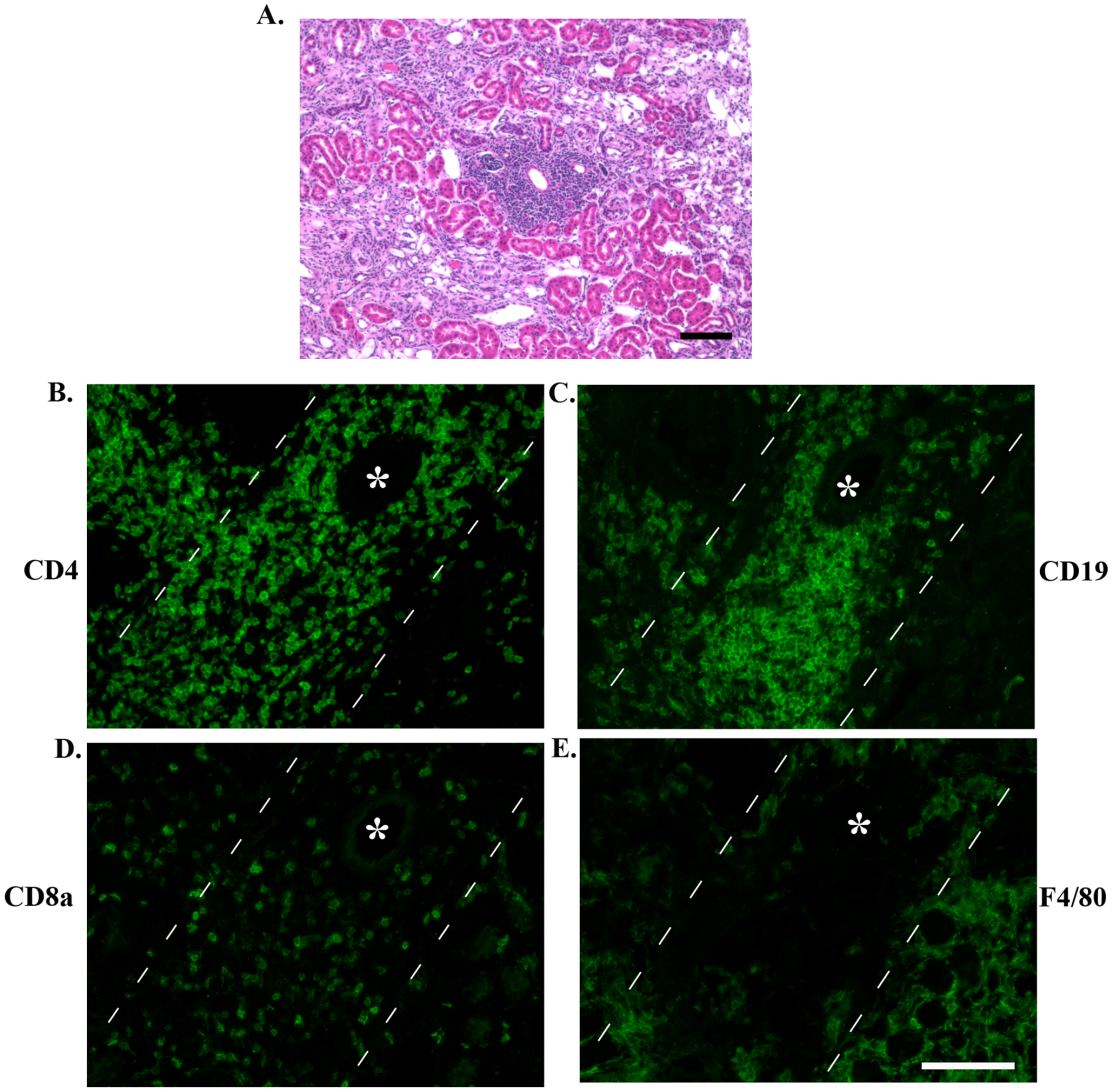
Discrete areas of leukocyte infiltrates surrounding the vasculature are only present in aged animals 6 weeks post-reperfusion. Representative H&E micrograph (A) showing a blood vessel surrounded by leukocytes. It is not the same vessel depicted in subsequent figures. Representative micrographs of serial sections indicate that CD4^+^ T lymphocytes (B) and CD19^+^ B lymphocytes (C) are the predominant immune cells in the area surrounding the blood vessel. Some CD8^+^ T lymphocytes also are present in lower numbers (D); however, F4/80^+^ macrophages do not co-localize with these leukocytes (E). *denotes the lumen of the blood vessel. Hatch marks indicate peripheral margin of the vascular sheath of denoted blood vessel. Bar = 100 microns.

A unique pathology in the aged kidneys 6 weeks post-reperfusion is leukocyte infiltration of the perivascular sheath of blood vessels (Figure 7A). This is not observed in young mice 6 weeks post-reperfusion or in sham kidneys (data not shown). The predominant cell types in the connective tissue sheath are CD4^+^ T lymphocytes and CD19^+^ B lymphocytes (Figure 7B+C). A few CD8^+^ T lymphocytes also are present (Figure 7D); however, F4/80^+^ macrophages are excluded (Figure 7E).

### Microvascular Loss is Greater in the Aged Kidney 6 Weeks Post-reperfusion

**Figure 8:**
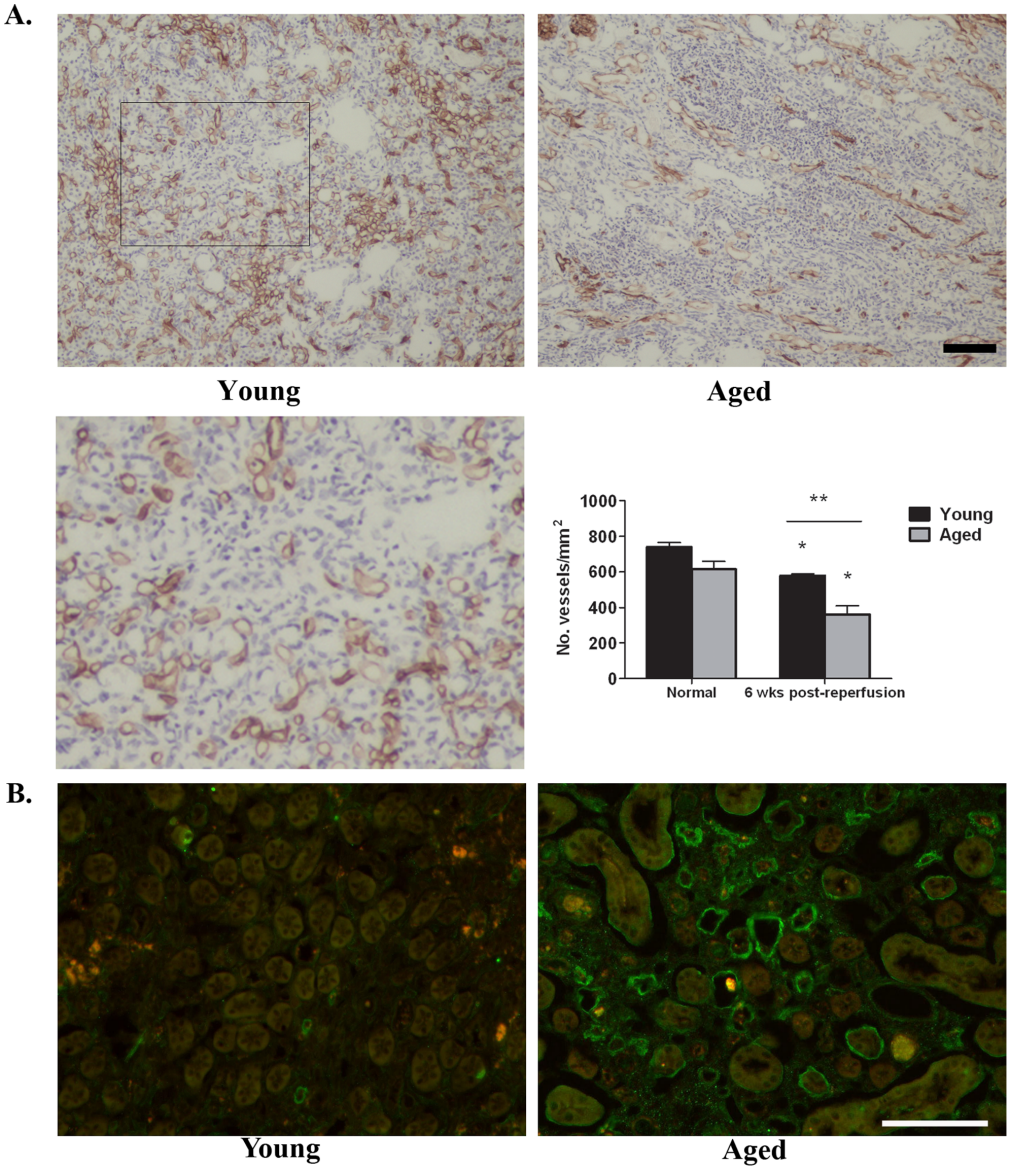
Microvascular loss and oxidative stress are greater in the aged kidney 6 weeks post-reperfusion. A. Representative micrographs of CD31-immunostained outer medulla from young and aged mice at 6 weeks post-reperfusion. The enlarged view (area indicated by box) highlights CD31^+^ endothelial cells lining the vasculature. Quantitation of CD31 immunostaining is shown in the graph. There is no difference between young and aged normal; however, vascular loss is greater in the aged kidney 6 weeks post-reperfusion. *p≤0.05 indicates a significant difference vs respective normal. **p≤0.05 indicates a significant difference between young and aged ischemia. B. Oxidative stress measured by nitro-tyrosine staining is greater in the outer medulla of aged kidneys 6 weeks post-reperfusion as compared to the young. Images were recorded with identical exposure settings. Bar = 100 microns.

Microvascular rarefaction is a long term outcome of AKI in both mice and rats [28], [29]. In young and aged normal kidneys (Figure 8A), CD31 immunostaining revealed no difference in microvascular density (young vs aged normal kidneys, 739±26 vessels/mm^2^ vs 615±44 vessels/mm^2^, respectively). However, microvessel density is significantly decreased in young kidneys 6 weeks post-reperfusion (577±10 vessels/mm^2^) compared to its respective normal (Figure 8A). Importantly, there is a significantly greater loss of microvessel density in the aged kidneys 6 weeks post-reperfusion (362±47 vessels/mm^2^) compared to the young 6 weeks (577±10 vessels/mm^2^; Figure 8A). Consistent with the recognized link between oxidative stress and microvascular loss [30] which promote fibrosis and inflammation following AKI [31], immunostaining for nitro-tyrosine is greater in the outer medulla of aged kidneys 6 weeks post-reperfusion (Figure 8B).

## Discussion

The present study clearly shows that mice, 46–49 weeks of age, have a prolonged injury and decreased survival rate following renal I/R compared to young mice that are approximately 8–10 weeks of age. Interestingly, no differences were observed in the loss of renal function at 24 hrs post-reperfusion, histological injury or cell death between the surviving young and aged mice. However, there was clearly a diminished proliferative response of the aged kidneys at both 24 and 48 hrs post-reperfusion. Furthermore, our data show that aged mice 6 weeks post-reperfusion have greater tubulo-interstitial fibrosis and leukocyte infiltration which correlates with increased p53 expression and the appearance of SA β-galactosidase positive cells in kidney tubules. Other long term outcomes 6 weeks post-reperfusion are greater vascular rarefaction and oxidative stress in the aged kidneys compared to the young. Importantly, no difference was measured in picrosirius stain, collagen III, fibronectin, collagen IV, leukocytes, p53 or percent SA β-galactosidase between young and aged sham or normal. Collectively, the data highlight differences in long term outcomes of AKI in aged versus young mice.

Following 30 min of bilateral renal ischemia, we observed a greater mortality rate in aged mice, reminiscent of human AKI [1], [32], [33] and similar to other studies in aged rodents [4], [18], [34]. The increased mortality with age is due to a sustained loss in renal function over several days, in contrast to the young, which normalize plasma creatinine and BUN within 48 hrs of reperfusion. Moreover, the degree of initial kidney injury 24 hrs post-reperfusion is similar between surviving young and aged mice based on the magnitude of the increase in plasma creatinine and BUN, histological injury scoring and apoptotic cell death. These data are in contrast to Ferenbach et al [18] and Schmitt et al [4] who demonstrated differences in renal function between young and aged mice; however, in these studies, shorter ischemia times were used and no substantial increase in either creatinine or BUN above baseline was measured in the young. Therefore, we believe we are inducing a severe ischemic injury with a 30 min bilateral clamp explaining why we are unable to measure significant differences between young and aged.

There are limitations to the use of plasma creatinine and BUN to evaluate renal function. Creatinine may be influenced by muscle mass, which decreases with age [35]. In our studies, both young and aged mice lost body weight immediately after renal I/R surgery, but over the course of the 6 week study, weight loss in the aged was approximately half that of the young (data not shown). Due to the inability to measure GFR post- reperfusion and potential limitations of creatinine and BUN measurements, we cannot rule out the possibility that differences in the initial injury between young and aged mice at 24 hrs post-reperfusion led to a prolonged recovery time, with consequently greater effects on fibrosis, leukocyte infiltration, cellular senescence and vascular loss. However, the injury and apoptotic scores 24 hrs post-reperfusion support the renal function measurement and despite the limitation to the use of creatinine and BUN, published studies in aged rodents continue to use them to measure renal function [4], [18].

Our data also suggest that the delayed recovery in kidney function is due in part to a diminished proliferative burst of the tubular epithelium, measured by PCNA staining at the 24 and 48 hrs post-reperfusion timepoint. Similarly, Schmitt et al. found that aged kidneys have a diminished proliferative capacity following I/R based on increased expression of zinc-α-(2)-glycoprotein (Zag), a potential suppressor of cell proliferation [4]. With age, structural and functional changes to the vasculature lead to diminished perfusion, lower epithelial stress resistance and decreases in renal mass [15], [16], [36]. These same changes in the aging kidney likely contribute to a delayed or incomplete recovery from AKI. In fact, patients over 65 years of age have a 28% higher risk of failing to completely recover kidney function after surviving AKI [3]. These data suggest that changes to the kidney that are inherent to aging may contribute to increased mortality following AKI while a diminished proliferative capacity will lead to incomplete recovery for those that survive AKI.

Incomplete repair of the kidney epithelium has also been shown to contribute to long term outcomes such as fibrosis [9], [12] that can lead to progressive organ dysfunction [11]. In fact, AKI is becoming more recognized as a cause of CKD [11], especially in the aging population [5], [17], [37]. However, little is known about the mechanism by which AKI progresses to CKD because relatively few studies have focused on the underlying pathophysiology and long term effects of renal I/R, which was the focus of our studies.

In addition to the greater fibrosis in aged kidneys 6 weeks post-reperfusion, our data show that p53 was significantly upregulated compared to the young. We did not evaluate the mechanism for increased p53 expression, but believe it to be a result of cellular stress, including hypoxia and DNA damage, that develop over the 6 week time period. Yang et al. [12] explained the increased p53 in models of ischemic AKI as due to induced activation of the ataxia telangiectasia-mutated (ATM) pathway.

Furthermore, p53 mediates kidney fibrosis of proximal tubular epithelial cells recovering from AKI via cell cycle arrest in G2/M. These arrested cells upregulate pro-fibrogenic growth factors such as transforming growth factor-β1 and connective tissue growth factor that in turn stimulate the proliferation of fibroblasts as well as collagen production by these cells. It is possible that elevated expression of p53 in aged kidneys post-reperfusion is inhibiting tubular epithelial cell proliferation via cell cycle arrest [12] and in this way is directly contributing to the greater fibrosis present 6 weeks post-reperfusion. In fact, elevated p53 correlated with enhanced deposition of cellular fibronectin as well as collagens III and IV in the aged. Expression of p53 has been linked to α-smooth muscle actin and fibrosis following in vitro nephrotoxic injury of tubular epithelial cells [25]. Interestingly, inhibition of p53 prevents cell cycle arrest and thus fibrosis [12].

Elevated p53 also has been linked to replicative and stress-induced senescence [38], [39]. The upregulation of p53 and p21 in combination with the appearance of SA β-galactosidase positive tubules supports the novel finding that cellular senescence is induced post-reperfusion, with greater numbers of senescent tubules in aged mice. We did not co-localize components of the p53-p21 pathway with SA β-galactosidase, a subject for future studies. However, Shimizu et al. [25] has demonstrated co-localization of p53 and β-galactosidase in the tubular epithelium in an in vivo model of chronic renal failure. There were very few β-galactosidase positive tubules in either young or aged normal kidneys, arguing that replicative senescence is unlikely to play a role as a senescence inducer post AKI. Rather, the senescence we detected is a result of cellular stress induced with I/R injury, i.e. stress-induced premature senescence [39], which has been linked to the development of interstitial fibrosis and tubular atrophy during kidney graft deterioration [40].

Associated with cellular senescence is the senescence associated secretory phenotype (SASP), responsible for the secretion of many factors including: growth factors, proteases, chemokines and cytokines. Although best studied in carcinogenesis, the most robustly induced and secreted among these factors are pro-inflammatory cytokines and chemokines [41]. It is unknown in our in vivo model whether senescent tubular epithelial cells are releasing pro-inflammatory mediators by acquiring the SASP and in this way contribute to the fibrosis that develops or whether senescence of the tubular epithelium is a consequence of fibrosis [38]. However, it is plausible that cellular senescence may be contributing to a pro-inflammatory microenvironment to promote fibrogenesis, which correlates with our observation of increased leukocyte infiltration and mRNA expression of MCP-1 and TNF-α.

Thus, another major contributor to fibrosis is inflammation. Our data in aged kidneys 6 weeks post-reperfusion indicate increased infiltration of F4/80^+^ cells and CD4^+^ T lymphocytes. Both cell types mediate persistent inflammation and fibrosis after AKI by secreting pro-fibrotic and pro-inflammatory cytokines such as MCP-1 and TNF-α leading to the development of CKD [26], [27], [42], [43], [44], [45], [46]. We did not examine macrophage phenotypes [47] in this study but have shown previously that M2 pro-fibrotic macrophages are the dominant macrophage phenotype at 5 weeks post-reperfusion in young mice [48]. Interestingly, in aged kidneys following AKI, CD4^+^ lymphocytes as well as CD8^+^ and CD19^+^ lymphocytes are found within the perivascular sheath, likely contributing to blood vessel loss and destruction.

Our data is the first to show that microvascular loss is significantly greater in aged kidneys 6 weeks post-reperfusion compared to the young. There is a reduction in microvessel density in both the young [28], [29] and aged mice compared to the respective normal. However, the decrease is more pronounced in aged kidneys following I/R. Decreased vascular repair and neovascularization are commonly associated with aging due to endothelial dysfunction, decreases in endothelial progenitor cell function and alterations in VEGF expression and stimulation [49], [50]. Each of these may play a role in the rarefaction observed in the aged animals following AKI. Diminished vascular repair may also contribute to a delayed or incomplete recovery of the tubular epithelium with subsequent deleterious effects on the tissue microenvironment. In aged kidneys 6 weeks post-reperfusion, inflammatory cell infiltrates containing F4/80^+^ and CD4^+^ leukocytes and deposition of fibronectin and collagens were significantly more robust than the young. Oxidative stress, indicated by nitrotyrosine staining, was also greater, and is known to be associated with kidney fibrosis that develops chronically following I/R injury [31]. Despite the enhanced evidence of progression to kidney dysfunction, no difference in renal function was measured between young and aged 6 weeks post-reperfusion.

In summary, our data suggest that the co-morbid model of renal I/R injury on a background of aging may be a more suitable pre-clinical model of AKI than the traditional model carried out in young animals. To date, all candidate therapeutics have failed in clinical trials, raising the question of whether the existing in vivo models of AKI [6] represent the patient population, who tend to be older, diabetic and/or hyperlipidemic [5], [8], [51]. Our data suggest the co-morbid model on a background of aging is more reflective of the patient population based on the increased mortality, incomplete recovery and increased histological evidence of progressive kidney dysfunction. We propose this model be used for pre-clinical testing of candidate therapeutics for drug development and be interrogated to identify and validate novel biological targets, which may be altered or differentially regulated compared to the young. Finally, the data presented here highlight the importance of complete tubular recovery following acute injury. Maintaining the proliferative and differentiation potential of the tubular epithelium has been an important therapeutic goal for the prevention of AKI but should be considered for long-term sequelae as well.
